# Individual Differences in the Rubber Hand Illusion Are Related to Sensory Suggestibility

**DOI:** 10.1371/journal.pone.0168489

**Published:** 2016-12-15

**Authors:** Angela Marotta, Michele Tinazzi, Clelia Cavedini, Massimiliano Zampini, Mirta Fiorio

**Affiliations:** 1 Department of Neurosciences, Biomedicine and Movement Sciences, University of Verona, Verona, Italy; 2 Department of Psychology and Cognitive Science, University of Trento, Rovereto, Italy; 3 Center for Mind/Brain Sciences, University of Trento, Trento, Italy; Birkbeck University of London, UNITED KINGDOM

## Abstract

In the rubber hand illusion (RHI), watching a rubber hand being stroked in synchrony with one’s own hidden hand may induce a sense of ownership over the rubber hand. The illusion relies on bottom-up multisensory integration of visual, tactile, and proprioceptive information, and on top-down processes through which the rubber hand is incorporated into pre-existing representations of the body. Although the degree of illusory experience varies largely across individuals, the factors influencing individual differences are unknown. We investigated whether sensory suggestibility might modulate susceptibility to the RHI. Sensory suggestibility is a personality trait related to how individuals react to sensory information. Because of its sensory nature, this trait could be relevant for studies using the RHI paradigm. Seventy healthy volunteers were classified by Sensory Suggestibility Scale (SSS) scores as having high or low suggestibility and assigned to either a high- (High-SSS) or a low-suggestibility (Low-SSS) group. Two components of the RHI were evaluated in synchronous and asynchronous stroking conditions: subjective experience of sense of ownership over the rubber hand via a 9-statement questionnaire, and proprioceptive drift as measured with a ruler. The High-SSS group was generally more susceptible to the subjective component; in the synchronous condition, they rated the statement assessing the sense of ownership higher than the Low-SSS group. The scores for this statement significantly correlated with the total SSS score, indicating that the higher the sensory suggestibility, the stronger the sense of ownership. No effect of sensory suggestibility on proprioceptive drift was observed, suggesting that the effect is specific for the subjective feeling of ownership. This study demonstrates that sensory suggestibility may contribute to participants’ experience of the illusion and should be considered when using the RHI paradigm.

## Introduction

The rubber hand illusion (RHI) is an experimental paradigm that investigates one of the fundamental components of self-consciousness: the sense of body ownership (i.e., the experience of the body as part of the self) [1–6]. In the RHI, watching a rubber hand being stroked in synchrony with one’s own hidden hand may induce a feeling of ownership over the rubber hand [5, 6]. It is thought that the illusion arises from bottom-up and top-down processes involved in multisensory integration [1], whereby the former are based on the temporal congruency between visual and tactile stimuli [1, 5, 7, 8] and the latter on the matching between the visual appearance of the rubber hand and a person’s internal model of the body [1, 9]. It seems that a combination of both bottom-up and top-down processes is necessary to create the illusion [1]. For example, the illusion is abolished when the object to be incorporated is morphologically different from a body part [9, 10] or when the rubber hand is positioned in an implausible posture [7] although visual and tactile stimuli are temporally congruent. Similarly, the illusion is also abolished when visual and tactile stimuli are temporally incongruent, even though the visual image of the rubber hand or its posture resemble the participant’s own hand [1–3, 5].

The illusion is commonly assessed by means of implicit and explicit measures that capture distinct, albeit related, aspects of the participant’s experience of the RHI [11]. The implicit measure is proprioceptive drift, which refers to a shift in the perceived position of one’s own hand towards the rubber hand [9, 12]. This measure is defined as implicit, in that participants are not asked to explicitly report how far their hand drifted toward the rubber hand, but rather to localize their hand before and after stroking. The drift is usually derived from the difference between the two measures [1, 13–15]. The explicit measure of the illusion refers to the subjective feeling of ownership [16] and it is obtained by directly asking participants to self-report their feelings via an *ad-hoc* questionnaire [5]. This measure is considered explicit, in that it captures the conscious experience that the rubber hand becomes part of the body.

Previous studies have revealed large variability on both measures of the RHI across individuals, which is likely rooted in specific dispositional traits [16–20]. However, despite the sensory nature of the RHI (i.e., visual, tactile, and proprioceptive), to our knowledge, no studies have investigated whether this variability could arise from an individual’s sensory suggestibility. This dispositional trait is closely related to how individuals react to ongoing sensory stimulation when surreptitiously influenced by another person [21–24]. For example, rubbing the tip of the tongue against the teeth does not evoke any taste perception. However, if verbally induced to believe that this action should elicit a sweet flavor, highly suggestible individuals are likely to perceive the suggested sensation, even though it is physiologically impossible.

A precondition for suggestibility to emerge is a context of ambiguity or uncertainty, in which an individual’s sensory perception can be easily influenced [22, 24]. With regard to the RHI, the artificial hand can be thought of as a suggestive stimulus [11] and the context appears to be characterized by the ambiguity and uncertainty of the source of information. Namely, synchronous stimulation of both the visible rubber hand and the hidden real hand creates perceptual ambiguity: tactile stimulation is felt on the real hand, but, at the same time, the visual information indicates that it is coming from the rubber hand. As an effect, the tactile and visual stimuli are integrated in a coherent, but physiologically impossible, percept: tactile and visual stimuli have a common source that is a visible rubber hand sensed as part of the body.

We investigated whether variability at the RHI can be partly explained by an individual’s sensory suggestibility. We predicted that highly sensory suggestible individuals would be more susceptible to experiencing the RHI than low sensory suggestible individuals. If sensory suggestibility generically affects an individual’s susceptibility to the illusion, we would find differences between highly and low suggestible individuals on both the explicit and implicit measures of the illusion. Conversely, finding a specific effect of sensory suggestibility on one of the two measures would demonstrate, for the first time, a specific role of this personality trait in selectively influencing the two components of the RHI.

## Materials and method

### Participants

Seventy naïve right-handed participants (36 women, mean age ± standard deviation: 34.99 ± 15.21 years) with no history of neurological or psychiatric illness volunteered for the study. Participants were recruited at the University of Verona and gave their written informed consent prior to the experiment. The study was approved by the local ethical committee of the Department of Neurosciences, Biomedicine and Movement Sciences, University of Verona, and was conducted according to the principles expressed in the Declaration of Helsinki.

### Procedure

All tests were performed in a single session lasting about one hour. Sensory suggestibility was assessed via the Sensory Suggestibility Scale (SSS; detailed description given below) [25], followed after a 10-minute interval by experiments with the RHI paradigm. The SSS score was computed at the end of the experiment to prevent any bias arising from the experimenter’s prior knowledge of the participant’s sensory suggestibility.

#### Sensory Suggestibility Scale

The SSS [25] score is a measure of indirect suggestibility in which the intention to influence remains concealed, with the participant unaware that her sensory suggestibility is going to be tested [23]. The SSS we used was similar to that used by Lund and colleagues [26]. It consisted of ten experimental and four control exercises [23, 26, 27] in which the experimenter induces tactile, visual, auditory or taste sensations in the participants through ad-hoc stimulation and verbal suggestions. In the experimental exercises, the suggested sensations are not physiologically plausible. In one exercise, for instance, the participant is asked to touch her right temple with the index and middle fingers and to count her heartbeat with eyes closed. The participant is then asked to remove her fingers from her temple and to rate on a scale from 0 (no sensation) to 4 (very strong sensation) whether and how vividly she can still perceive her heartbeat on the index and middle fingertips. Although the sensation of heartbeat on the fingertips is physiologically implausible, highly sensory suggestible participants will usually rate with high scores their subjective experience, meaning that the suggested sensation (e.g., feeling heartbeat on the fingertips) has been vividly perceived. Conversely, low sensory suggestible participants are more likely to give their experience low scores (e.g., no sensation of heartbeat on the fingertips). Assessment with the SSS is made more plausible when four control exercises are included that effectively evoke the suggested sensation. In one exercise, for example, the participant is asked to close one ear with her index finger and rate the sensation of hearing an ocean-like sound. The scores for this control exercise are typically high, since the suggested sensation effectively occurs. In our study, we administered two out of four control exercises in order to shorten the total duration of the experiment. The total SSS score (range 0–40) is computed by summing the scores recorded for the experimental exercises.

In order to specifically investigate the relationship between the degree of sensory suggestibility and participants’ susceptibility to the RHI, we split the sample according to the median of the total SSS score (n = 70, median = 13). Participants with a total SSS score < 13 were assigned to the Low-SSS group (n = 30, 16 women, mean age ± SD 38.23 ± 16.05 years, mean SSS score ± SD 8.27 ± 2.29) and those with a total SSS score > 13 were assigned to the High-SSS group (n = 31, 16 women, mean age ± SD 31.55 ± 13.06 years, mean SSS score ± SD 18.42 ± 3.40). Participants with a total SSS score of 13 (median) (n = 9) were excluded from further analysis. The final sample was composed of 61 participants.

#### Rubber hand illusion paradigm

For this study, we used a modified version of the original RHI paradigm devised by Botvinick and Cohen [5]. The experimental set-up consisted of two adjacent boxes (18x29 cm each) placed on a table in front of the participant, with the participant’s hand inside one box and the other hand resting on the leg. A realistic artificial hand (i.e., left or right) was placed in an anatomically plausible posture inside the other box. The artificial hand was 20 cm away from the participant’s hand (e.g., distance between the two index fingertips). During stimulation, the artificial hand was visible to the participant and her own hand was hidden from view by covering the box with a black drape. The experimenter, concealed behind a panel, used two small paintbrushes to synchronously or asynchronously stroke the same fingers of the visible artificial hand and of the participant’s hidden hand. The brush strokes were applied to the index, middle or ring finger of both hands in random order at approximately 1 Hz. The stimulation session lasted 2 minutes, during which the participant was asked to watch the artificial hand and focus on her feelings. No further information was given. The RHI paradigm was applied to both the right and the left hand in a counterbalanced order across participants. The type of stimulation (i.e., synchronous or asynchronous) was also delivered in a counterbalanced order. One trial in each condition was performed (right hand synchronous stimulation, right hand asynchronous stimulation, left hand synchronous stimulation, left hand asynchronous stimulation).

At the end of each trial, the participants completed a 9-statement questionnaire [5] that measures the explicit component of the RHI. Each statement describes a specific perceptual effect that can be more or less vividly experienced during stimulation. Participants rated their agreement or disagreement with each statement on a numeric rating scale ranging from 0 ("I totally disagree") to 10 ("I totally agree"). The first three statements (“Illusion-related statements”) are most frequently endorsed after synchronous stimulation and are considered as being the ones most sensitive to capture the illusory embodiment of the rubber hand [11]. In detail, statement 1 (S1) refers to an illusory localization of touch on the rubber hand, S2 refers to a causal link between vision and touch, and S3 is more directly related to an illusory feeling of ownership. Statements 4 to 9 are considered control items (“Control statements”) because they refer to perceptual effects that are unrelated to the illusory embodiment of the rubber hand [7, 16]. These statements allow to control for a participant’s compliance with the task [5, 7], in that they usually receive lower agreement scores than the illusion-related statements. All nine statements are listed in Table 1.

**Table 1:** Illusion-related and control statements. Statistical results of post-hoc comparisons between synchronous and asynchronous conditions in the two groups and in both hands (Wilcoxon signed-rank test).

	Synchronous vs Asynchronous			
	High suggestibility group		Low suggestibility group	
	Right hand	Left hand	Right hand	Left hand
**Illusion-related statements**				
1. It seemed as if I felt the paintbrushes touching my finger where I saw the rubber hand being touched.	Z = -2.829	Z = -4.134	Z = -4.091	Z = -4.471
	*P* = 0.005	*P* < 0.001	*P* < 0.001	*P* < 0.001
2. It seemed like the touch I felt was caused by the paintbrushes touching the rubber hand.	Z = -3.585	Z = -4.560	Z = -4.028	Z = -4.413
	*P* < 0.001	*P* < 0.001	*P* < 0.001	*P* < 0.001
3. I felt as if the rubber hand was my own hand.	Z = -4.337	Z = -4.312	Z = -4.036	Z = -3.660
	*P* < 0.001	*P* < 0.001	*P* < 0.001	*P* < 0.001
**Control statements**				
4. It felt like my hand was drifting toward the rubber hand.	Z = -3.463	Z = -3.431	Z = -2.767	Z = -2.438
	*P* = 0.001	*P* = 0.001	*P* = 0.006	*P* = 0.015
5. It seemed like I might have more than one hand or arm.	Z = -2.080	Z = -1.932	Z = -2.782	Z = -1.481
	*P* = 0.038	*P* = 0.053	*P* = 0.005	*P* = 0.139
6. It seemed as if the touch I was feeling came from somewhere between my own hand and the rubber hand.	Z = -2.147	Z = -3.103	Z = -2.245	Z = -2.263
	*P* = 0.032	*P* = 0.002	*P* = 0.025	*P* = 0.024
7. It felt like my hand was turning rubbery.	Z = -2.134	Z = -2.695	Z = -2.523	Z = -0.789
	*P* = 0.033	*P* = 0.007	*P* = 0.012	*P* = 0.430
8. It appeared as if the rubber hand were drifting towards my hand.	Z = -1.560	Z = -2.389	Z = -0.120	Z = -1.980
	*P* = 0.119	*P* = 0.017	*P* = 0. 904	*P* = 0.048
9. The rubber hand began to resemble my own hand.	Z = -2.545	Z = -3.083	Z = -3.113	Z = -3.275
	*P* = 0.011	*P* = 0.002	*P* = 0.002	*P* = 0.001

We used proprioceptive drift measurement as an implicit measure of the illusion. Proprioceptive drift can be defined as a recalibration of the perceived position of the participant’s own hand toward the artificial hand. Before and after stimulation, a ruler was placed on top of the box and the participant was asked to verbally report the number corresponding to the perceived position of her index finger. The position of the ruler was changed within and between conditions in order to control for response biases. During this part of the experiment, both the rubber hand and the participant’s hand were hidden from view. Proprioceptive drift was calculated by subtracting the estimations of finger position obtained before and after stimulation. Previous studies have demonstrated a shift in the perceived position of the participant’s hand towards the rubber hand after synchronous but not after asynchronous stimulation [1, 5, 9].

### Data analyses

Preliminary analyses with the t-test for independent samples and the chi-square test showed that the two groups were comparable for age and gender, respectively. Questionnaire scores and measurement of proprioceptive drift in all conditions (synchronous and asynchronous stroking of the left and the right hand) were inspected to exclude potential outliers (i.e., values 3×SD above or below the mean value of the whole sample). All data were assessed for normal distribution using the Shapiro-Wilk test (p> 0.05). Since the data were not normally distributed, non-parametric tests were applied. The Friedman test was used to compare, within each group, the statement ratings across conditions (synchronous and asynchronous stroking) for both hands (right and left). The self-reported rating of each statement was analysed separately. For the post-hoc pairwise comparisons, the Wilcoxon signed-rank test with appropriate Bonferroni correction was applied as necessary. Group differences in each condition and for each hand were analyzed using the Mann-Whitney U test. Spearman’s coefficient of correlation was used to test the relation between the total SSS score and the RHI measures (i.e., questionnaire and proprioceptive drift) obtained in each condition and for each hand. Statistical significance was set at p ≤ 0.05. All data are expressed as means ± standard error (SE).

## Results

The two groups were comparable for age (t(59) = 1.790, *P* = 0.079) and gender distribution (χ^2^ = 0.018, *P* = 0.893). We found outliers for some of the questionnaire control statements: one participant was an outlier for S4, one for S5, one for S6, and two participants were outliers for S8. Analyses of these control statements were run after having removed the outliers. Two participants were outliers for proprioceptive drift; their data were also removed from the analysis of the drift.

### Questionnaire

#### Illusion-related statements

In the High-SSS group, the Friedman test was significant for all three illusion-related statements (S1: χ^2^ (3) = 32.351, *P* < 0.001; S2: χ^2^ (3) = 56.414, *P* < 0.001; S3: χ^2^(3) = 43.722, *P* < 0.001). Post-hoc comparisons (critical p ≤ 0.025 after Bonferroni correction) showed that the ratings given to all three statements (S1, S2, S3) after synchronous stimulation were significantly higher than those given after asynchronous stimulation (see Table 2 for mean values and standard errors), for both the right and the left hand (for all comparisons, Z < -2.829, *P* < 0.005, see Tables 1 and 2 for statistical details). Similar scores were observed for the Low-SSS group, where the Friedman test was significant for all three illusion-related statements (S1: χ^2^ (3) = 52.340, *P* < 0.001; S2: χ^2^ (3) = 54.013, *P* < 0.001; S3: χ^2^ (3) = 39.743, *P* < 0.001) due to the higher ratings given after synchronous than asynchronous stimulation of both the right and the left hand (for all comparisons, Z < - 3.660, *P* < 0.001, see Tables 1 and 2 for statistical details).

**Table 2:** Mean (± standard error) scores calculated for each illusion-related and control statement in the two groups and for both hands.

	High suggestibility group				Low suggestibility group			
	Right hand		Left hand		Right hand		Left hand	
	Synchronous	Asynchronous	Synchronous	Asynchronous	Synchronous	Asynchronous	Synchronous	Asynchronous
**Illusion-related statements**								
S1	6.90 (0.63)	4.26 (0.70)	8.32 (0.49)	3.68 (0.69)	7.00 (0.65)	2.43 (0.60)	6.97 (0.58)	2.27 (0.56)
S2	7.23 (0.59)	3.58 (0.63)	7.84 (0.51)	2.55 (0.50)	6.633 (0.63)	2.57 (0.62)	7.30 (0.55)	2.63 (0.67)
S3	7.39 (0.54)	3.19 (0.63)	8.13 (0.46)	3.16 (0.65)	4.97 (0.73)	1.93 (0.46)	5.40 (0.69)	2.43 (0.65)
**Control statements**								
S4	3.53 (0.55)	1.63 (0.37)	3.93 (0.58)	1.60 (0.37)	2.07 (0.55)	0.83 (0.35)	2.03 (0.53)	0.80 (0.32)
S5	2.80 (0.55)	1.53 (0.44)	3.57 (0.60)	2.33 (0.54)	2.47 (0.59)	0.80 (0.32)	2.20 (0.61)	1.40 (0.48)
S6	2.90 (0.56)	1.37 (0.32)	2.90 (0.56)	1.37 (0.32)	2.30 (0.64)	0.83 (0.31)	1.57 (0.53)	0.73 (0.28)
S7	4.87 (0.66)	2.77 (0.64)	4.87 (0.66)	2.77 (0.64)	2.60 (0.58)	1.17 (0.38)	2.03 (0.58)	1.83 (0.53)
S8	2.43 (0.52)	1.37 (0.45)	2.43 (0.52)	1.37 (0.42)	0.93 (0.35)	0.90 (0.33)	1.45 (0.49)	0.76 (0.32)
S9	5.16 (0.64)	3.26 (0.66)	5.48 (0.63)	3.26 (0.66)	3.10 (0.60)	1.33 (0.43)	3.70 (0.64)	3.27 (0.60)

**Fig 1:**
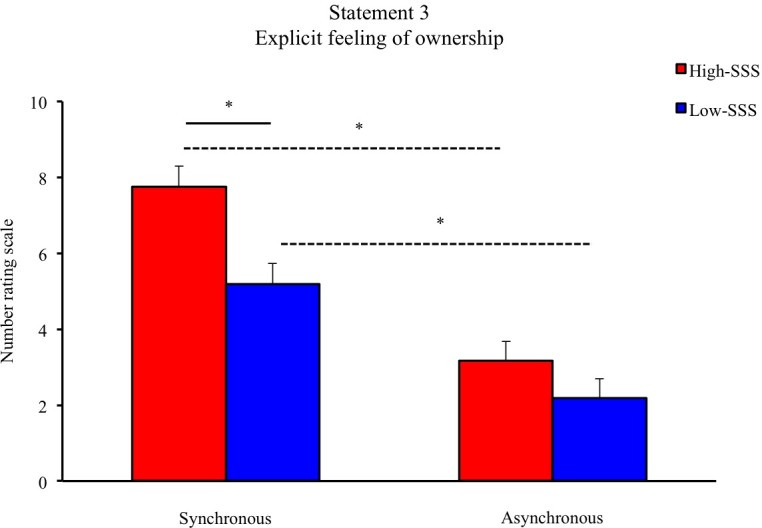
Mean scores for S3 related to an explicit feeling of ownership after synchronous and asynchronous stroking. The solid line shows a significant difference in scores between the two groups in the synchronous condition. The dashed lines indicate significant differences between synchronous and asynchronous conditions within each group. In both groups, the experience of illusion was rated higher after synchronous than after asynchronous stimulation. Error bars represent standard errors.

The High-SSS group rated S1 higher after right hand synchronous stimulation (8.32 ± 0.492) than after left hand synchronous stimulation (6.90 ± 0.633) (Z = - 2.527, *P* = 0.012). On comparison of the two groups (High-SSS vs. Low-SSS) across conditions for both the right and the left hand, the High-SSS group rated S3 significantly higher after synchronous, but not after asynchronous, stimulation, of both the right (Z = -2.378, *P* = 0.017) and the left hand (Z = -2.378, *P* = 0.002) (Fig 1). We observed no difference in the other illusion-related statements or between hands between the two groups.

**Fig 2:**
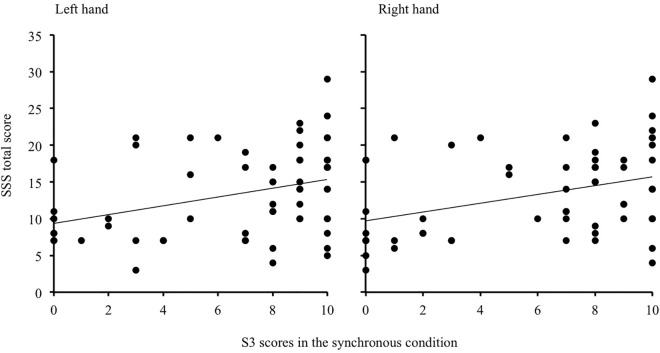
Correlation between S3 scores in the synchronous condition and the total SSS score. The graph shows Spearman’s correlation between the total SSS score (y-axis) and the explicit feeling of ownership (S3) after synchronous stimulation (x-axis) of the left and the right hand. The higher the degree of sensory suggestibility, the stronger the feeling of ownership of the rubber hand.

S3 specifically and directly assesses the feeling of ownership over the rubber hand, which requires synchronous stroking to occur. Scores for S3 correlated with the total SSS score for both the right (rho = 0.352, *P* = 0.005) and the left hand (rho = 0.340, *P* = 0.007) only in the synchronous condition (Fig 2). This finding further supports a link between the illusory feeling of ownership and sensory suggestibility by demonstrating that the intensity of feeling ownership of the rubber hand increases with the degree of sensory suggestibility, specifically when the illusion is expected to arise (i.e., after synchronous stimulation).

Additional positive correlations were observed between S1 and S2 scores for the right hand, but only in the asynchronous condition (S1: rho = 0.271, *P* = 0.034; S2: rho = 0.282, *P* = 0.027).

#### Control statements

In the High-SSS group, the Friedman test was significant for all six control statements (S4: χ^2^(3) = 21.772, *P* < 0.001; S5: χ^2^(3) = 11.050, *P* = 0.011; S6: χ^2^(3) = 15.645, *P* = 0.001; S7: χ^2^(3) = 8.898, *P* = 0.031; S8: χ^2^(3) = 11.287, *P* = 0.010; S9: χ^2^(3) = 20.250, *P* < 0.001). Post-hoc comparisons (critical p ≤ 0.025 after Bonferroni correction) showed significant differences between synchronous and asynchronous stimulation of the right hand for S4 and S9 scores (all comparisons, Z < -2.545, P< 0.011) and of the left hand for all statements (except S5) (all comparisons, Z < -2.389, *P* < 0.017). No differences between conditions (right or left hand stimulation) were noted for S5. We observed no differences between the two hands for any of the control statements.

In the Low-SSS group, the Friedman test was significant for the majority of the control statements (S4: χ^2^(3) = 16.458, *P* = 0.001; S5: χ^2^(3) = 12.199, *P* = 0.005; S6: χ^2^(3) = 12.838, *P* = 0.005; S7: χ^2^(3) = 8.756, *P* = 0.033; S9: χ^2^(3) = 21.396, *P* < 0.001). Post-hoc comparisons showed that the scores for all statements (except S8) were higher after synchronous than after asynchronous stimulation of the right hand (all comparisons, Z < -2.245, *P* < 0.025). Moreover, scores for S4 and S6 were higher after synchronous than after asynchronous stimulation of the left hand (all comparisons, Z < -2.263, *P* < 0.024). Finally, post-hoc comparisons also showed that the scores for S9 were higher after asynchronous stimulation of the left hand as compared to the right hand (Z = -2.704, *P* = 0.007).

The Mann-Whitney U tests revealed significant differences between groups owing to the higher scores in the High-SSS group, as compared to the Low-SSS, for S4, S7, and S9 in both stimulation conditions and hands (all comparisons, Z < -1.978, *P* < 0.048).

Differences between groups were also found for S6 scores after synchronous stimulation of the left hand (Z = -2.170, *P* = 0.030) and after asynchronous stimulation of the right hand (Z = -2.448, *P* = 0.014). As compared to the Low-SSS group, the High-SSS group rated S5 higher after asynchronous stimulation of the left hand (Z = -1.994, *P* = 0.046) and S8 higher after synchronous stimulation of the right hand (Z = -2.561, *P* = 0.010). Significant correlations were found between the total SSS score and scores for S4, S7, S8, and S9 after both synchronous and asynchronous stimulation of the right hand (all, rho ≤ 0.435, *P* ≤ 0.019) and for S6 in the asynchronous condition (rho = 0.400, *P* = 0.002). The total SSS score significantly correlated with S4, S6, and S7 scores after both synchronous and asynchronous stimulation of the left hand (all, rho ≤ 0.310, *P* ≤ 0.026), and with S8 scores in the synchronous condition (rho = 0.269, *P* = 0.039).

#### Proprioceptive drift

In the High-SSS group, the Friedman test was significant (χ^2^(3) = 9.715, *P* = 0.021). Post-hoc comparisons showed that this effect was due to greater proprioceptive drift after synchronous than after asynchronous stimulation of the left hand (synchronous, 2.393 ± 0.477 cm; asynchronous, 0.655 ± 0.494 cm) (Z = -2.878, *P* = 0.004). Similarly, in the Low-SSS group, the Friedman test was significant (χ^2^(3) = 8.960, *P* = 0.030). Again, post-hoc comparisons showed that this effect was due to greater proprioceptive drift after synchronous than after asynchronous stimulation of the left hand (synchronous, 2.133 ± 0.313 cm; asynchronous, 0.833 ± 0.419 cm) (Z = -2.962, *P* = 0.003). We observed no significant differences between synchronous or asynchronous stimulation of the right hand in either group. No differences were found between the left and the right hand in either of the two conditions or between the two groups. Proprioceptive drift was similar in both of the conditions, the hands, and the groups. The total SSS score did not significantly correlate with proprioceptive drift.

## Discussion

Here we investigated whether sensory suggestibility might influence susceptibility to the RHI. Our results clearly show that the explicit component of the RHI is modulated by sensory suggestibility and that highly suggestible individuals experience a stronger illusory sense of ownership than low suggestible individuals do.

Certain personality traits reportedly account for individual variability at the RHI [18–20], including paranoid ideation, psychoticism predisposition, and interpersonal sensitivity [18–20], which are not directly related to sensory perception, although the RHI is related to specific types of sensory stimulation (i.e., visual, tactile, and proprioceptive). The current study sheds new light on the factors influencing individual susceptibility to the RHI. Our findings demonstrate, for the first time, that sensory suggestibility, a personality trait closely related to perceptual experiences, selectively influences the explicit feeling of ownership of the rubber hand, but not implicit proprioceptive drift.

One could argue that highly suggestible individuals are generally more compliant and tend to rate questionnaire statements higher [11]. Although we cannot completely exclude this factor, we believe that compliance alone cannot explain our findings. Namely, if the results were solely due to compliance, we should have found an effect of suggestibility independent of the stimulation condition on all the statements. Instead, we found differences between highly and low suggestible individuals for S3 selectively after synchronous and not asynchronous stimulation. Among the different perceptual effects of the RHI, S3 relates specifically to feeling ownership of the rubber hand (“I felt as if the rubber hand were my own hand”) [5]. Finding a difference between groups after synchronous stimulation rules out a general effect of suggestibility and points to a specific effect on feeling ownership. This observation may be corroborated by future studies investigating the illusory experience of feeling body ownership, for example, by using several variants of S3 (e.g., “It felt like the rubber hand was part of my body”; “It felt like the rubber hand belonged to me”; or “It felt like the rubber hand was part of my body”) [28, 29].

The ratings of the other two statements related to embodiment (i.e., incorporating the artificial hand into an internal body model), like S1 (“It seemed as if I felt the paintbrushes touching my finger where I saw the rubber hand being touched”) and S2 (“It seemed like the touch I felt was caused by the paintbrushes touching the rubber hand”) did not differ significantly between the two groups, hinting, again, at a specific effect for S3. A principal components analysis study has shown that S1 and S2 are more closely related to the localization of touch, whereas S3 is solely related to the feeling of ownership. These effects have been found to dissociate: feeling ownership does not imply localizing touch on the rubber hand and *vice versa* [28]. Moreover, as posited within a previous theoretical framework, feeling ownership and localizing touch refer to two different processes of embodiment [9]: localizing tactile sensation on the rubber hand (S1 and S2) involves bottom-up processes based on visual-tactile integration, whereas feeling ownership over the rubber hand (S3) requires additive top-down processes based on prior knowledge of what the body looks like [1, 28]. Hence, we speculate that sensory suggestibility more likely interacts with top-down rather than with bottom-up processes involved in the RHI and therefore predisposes highly suggestible individuals to more readily experience the artificial hand as being a part of their body.

With regard to proprioceptive drift, we noted a significant difference between synchronous and asynchronous stimulation of the left hand. This might be related to the relevance of the right hemisphere in the RHI [30, 31]. Interestingly, we observed no effect of sensory suggestibility on proprioceptive drift. Previous studies have shown a shift in the perceived position of the participant’s hand towards the rubber hand even after asynchronous stroking [32] or with a rubber hand rotated 180 degrees [15]. Although proprioceptive drift was present in these conditions, the participants did not report a feeling of ownership over the rubber hand, indicating a dissociation between these two components of the RHI [12, 15, 32, 33]. Owing to this dissociation, proprioceptive drift is thought to arise from multisensory integration processes that do not necessarily require the rubber hand to be felt as part of the body [12, 15, 32, 33]. In our study, the lack of effect of sensory suggestibility on proprioceptive drift might indicate that this personality trait does not affect the multisensory processes underlying the drift (i.e., visuo-proprioceptive integration). Conversely, the specific effect of sensory suggestibility on the feeling of ownership hints at modulation of the top-down matching processes through which the rubber hand is admitted to be part of a pre-existing internal model of the body.

Our observations contrast with a recent study [11] in which proprioceptive drift was found to positively correlate with another type of suggestibility—hypnotic suggestibility (i.e., the ability to experience suggested sensations, emotions, and thoughts during hypnotic induction) [11]. Sensory and hypnotic suggestibility involve different cognitive processes, however: the former requires an imagery ability related to a non-existent, but suggested, sensation [23], whereas the latter also involves heightened attentional focus and the ability to ignore sources of interference during hypnotic induction [11]. These two types of suggestibility may differently influence the two components of the RHI. In this regard, previous studies demonstrated that the ability to imagine the movement of a virtual hand is related to an explicit feeling of ownership, but not to proprioceptive drift [34, 35]. Based on our findings, we speculate that the higher imagery ability underlying sensory suggestibility could specifically facilitate incorporation of the rubber hand into an internal model of the body, thus inducing an explicit feeling of ownership. Conversely, as suggested in a recent study, the attentional ability underlying hypnotic suggestibility may specifically facilitate the visuo-proprioceptive integration at the basis of implicit proprioceptive drift [11].

Finally, we also found an unspecific effect of sensory suggestibility on some control statements. The differences for S4, S7, and S9 scores between the two groups, independent of the type of stimulation (synchronous or asynchronous) and of the hand stimulated, suggest that highly sensory suggestible individuals are also more prone than low suggestible ones to feel other perceptual effects at the RHI, such as feeling their own hand drifting towards the rubber hand (S4) and perceiving similarity between their own and the rubber hand (S7 and S9). Interestingly, the sensations captured by some of these statements, like S7 (“It felt like my hand was turning rubbery”) and S9 (“The rubber hand began to resemble my own hand”), have been associated with embodiment of the rubber hand [28]. These findings could be interpreted as a general tendency of highly suggestible individuals to respond affirmatively to all the statements. A difference between the two groups was found also for S8, which refers to a perceived shift of the rubber hand towards the participant’s own hand. It should be noted, however, that the scores for this control statement were very low, suggesting a general disagreement.

This study has several limitations. First, while the SSS has been used in previous studies [23, 26], to our knowledge, its test-retest reliability has not yet been examined. If the SSS scores are not stable over time, they might correlate with the RHI in one session but not in another, thus undermining the strength of the current findings. Second, to our knowledge, little is known about the factors contributing to sensory suggestibility. Some evidence suggests differences related to gender and age [36]. In this regard, one study found that sensory suggestibility is higher when subjects are tested by an experimenter of the same gender [37]. A future area of focus would be to consider this variable also in the context of the RHI. Third, all our participants were naïve to both the RHI task and the SSS tool. Future investigations are needed to determine whether the association between sensory suggestibility and the RHI would hold also in non-naïve subjects. This would yield valuable information on the strength of the link between sensory suggestibility and an individual’s susceptibility to the RHI.

## Conclusions

This study demonstrates for the first time the role of sensory suggestibility in influencing an individual’s susceptibility to illusory experience induced by the RHI. We found that highly suggestible individuals were more likely to agree with many of the questionnaire statements than low suggestible individuals. However, the explicit feeling of ownership of the rubber hand was specifically influenced by sensory suggestibility during synchronous stimulation. Other main perceptual RHI effects, like the illusory localization of touch on the rubber hand and proprioceptive drift were not modulated by sensory suggestibility. Since the feeling of ownership is mainly driven by top-down processes, we speculate that sensory suggestibility influences the subjective experience of feeling ownership by interacting with the top-down processes underlying the RHI through the enhancement of the salience of visual stimuli.
